# Hypoxia-Induced Changes in DNA Methylation Alter RASAL1 and TGFβ1 Expression in Human Trabecular Meshwork Cells

**DOI:** 10.1371/journal.pone.0153354

**Published:** 2016-04-28

**Authors:** Fiona McDonnell, Mustapha Irnaten, Abbot F. Clark, Colm J. O’Brien, Deborah M. Wallace

**Affiliations:** 1 School of Medicine and Medical Science, University College Dublin, Dublin 4, Ireland; 2 Dept. Cell Biology & Immunology and the North Texas Eye Research Institute, U. North Texas Health Science Center, Ft. Worth, Texas, United States of America; 3 Dept. Ophthalmology, Mater Misericordiae University Hospital, Dublin 7, Ireland; University of Hong Kong, HONG KONG

## Abstract

**Purpose:**

Fibrosis and a hypoxic environment are associated with the trabecular meshwork (TM) region in the blinding disease glaucoma. Hypoxia has been shown to alter DNA methylation, an epigenetic mechanism involved in regulating gene expression such as the pro-fibrotic transforming growth factor (TGF) β1 and the anti-fibrotic Ras protein activator like 1 (RASAL1). The purpose of this study was to compare DNA methylation levels, and the expression of TGFβ1 and RASAL1 in primary human normal (NTM) with glaucomatous (GTM) cells and in NTM cells under hypoxic conditions.

**Methods:**

Global DNA methylation was assessed by ELISA in cultured age-matched NTM and GTM cells. qPCR was conducted for TGFβ1, collagen 1α1 (COL1A1), and RASAL1 expression. Western immunoblotting was used to determine protein expression. For hypoxia experiments, NTM cells were cultured in a 1%O_2_, 5%CO_2_ and 37°C environment. NTM and GTM cells were treated with TGFβ1 (10ng/ml) and the methylation inhibitor 5-azacytidine (5-aza) (0.5μM) respectively to determine their effects on DNA Methyltransferase 1 (DNMT1) and RASAL1 expression.

**Results:**

We found increased DNA methylation, increased TGFβ1 expression and decreased RASAL1 expression in GTM cells compared to NTM cells. Similar results were obtained in NTM cells under hypoxic conditions. TGFβ1 treatment increased DNMT1 and COL1A1, and decreased RASAL1 expression in NTM cells. 5-aza treatment decreased DNMT1, TGFβ1 and COL1A1 expression, and increased RASAL1 expression in GTM cells.

**Conclusions:**

TGFβ1 and RASAL1 expression, global DNA methylation, and expression of associated methylation enzymes were altered between NTM and GTM cells. We found that hypoxia in NTM cells induced similar results to the GTM cells. Furthermore, DNA methylation, TGFβ1 and RASAL1 appear to have an interacting relationship that may play a role in driving pro-fibrotic disease progression in the glaucomatous TM.

## Introduction

Glaucoma is an optic neuropathy that affects approximately 60 million people worldwide[1]. In glaucoma, the retinal ganglion cell (RGC) axons are irreversibly lost in a way that contributes to the visual field loss pattern seen in patients[2]. Some of the factors that contribute to the disease include: increased intraocular pressure (IOP), age, genetic mutations, and reduced ocular perfusion pressure (OPP)[3–7]. Under normal circumstances, there is a process of physiological wound healing in the body; however, in some diseases, this wound healing becomes uncontrolled leading to connective tissue fibrosis[8, 9]. In glaucoma, fibrosis occurs as a build-up of extracellular matrix (ECM) materials in the trabecular meshwork (TM) at the anterior of the eye[10–12], and in the lamina cribrosa (LC) at the optic nerve head (ONH)[13–15]. This mechanism of fibrosis plays a role in the disease progression; ECM materials build up in the TM and the fluid within the eye, the aqueous humor (AH), cannot easily exit via its normal pathway, and the pressure within the eye subsequently increases. This increase in IOP is one of the main risk factors associated with the development and progression of glaucoma[4, 16] and is the only target for therapies in clinical use[17]. Following the increased IOP, structural damage occurs at the optic nerve head, which is associated with the loss of RGC axons and the loss of vision in glaucoma[18, 19].

There are a number of profibrotic factors found to be increased in the AH and TM of glaucomatous eyes. These include transforming growth factor β2 (TGFβ2) in primary open angle glaucoma (POAG)[20] and TGFβ1[21] and connective tissue growth factor (CTGF) in pseudoexfoliation glaucoma (PXFG)[22]. These factors have been shown to be involved in ECM production[23–25], and as TGFβ2 is present in the AH of human eyes[20], it is possible that it drives the production of ECM in the TM. As previous work from our group has shown, there are increased levels of TGFβ1 in the LC cells of POAG eyes[26] and increased levels of CTGF in the AH of PXFG eyes, affecting the TM[22]. TGFβ1 has been shown to be the primary isoform in PXFG, and the main site of pseudoexfoliation syndrome deposits in glaucoma occur in the TM region[27]. Further, it has been shown in a number of fibrotic diseases that TGFβ plays a role in mediating fibrosis and causes an increase in ECM deposition[28–30]. Studies show that the same is true in the process of glaucoma—increased levels of TGFβ lead to increased ECM deposition in the TM and LC of glaucomatous eyes[30]. In an attempt to combat fibrosis, a number of therapeutic approaches have been studied. SB431542 is an inhibitor of the ALK5 receptor (TGFβ type I receptor) and therefore acts as an inhibitor of TGFβ signalling[31]. This inhibitor has also been shown to downregulate TGFβ-induced ECM genes in TM cells[30, 32]. Work by our laboratory has shown that a humanized monoclonal anti-CTGF antibody FG-3019 was able to effectively block ECM production as shown by a significant reduction in the expression of profibrotic genes[33], in LC and TM cells treated with AH samples from pseudoexfoliation glaucoma (PXFG), primary open angle glaucoma (POAG), and hydrogen peroxide.

There is a further cellular mechanism by which fibrosis may be regulated, through epigenetics. Epigenetics is the study of heritable changes in gene function caused by mechanisms other than changes in the underlying DNA sequence[34]. It involves DNA methylation[35] and histone modifications including acetylation/deacetylation and methylation[36]. Micro RNAs (miRNAs) have been established as regulators of fibrosis in cardiac, kidney and lung fibrosis [37–39]. It has recently been demonstrated that epigenetic mechanisms may play a role in the regulation of miRNAs and that miRNAs use epigenetic mechanisms to mediate their downstream effects in cardiovascular disease and pulmonary fibrosis [40–42]. It has been proposed that these epigenetic processes play a role in the progression of fibrosis in a number of diseases[43–45]. Enzymes that contribute to DNA methylation include DNA methyltransferases (DNMTs)[46, 47]–DNMT1 is involved in maintaining DNA methylation[48] and DNMT3A is involved in *de novo* methylation[48]. The transcriptional repressor/activator Methyl CPG binding protein 2 (MeCP2) binds to methylated DNA and recruits transcription factors to regulate gene expression[49, 50].

It has been previously observed that fibroblasts isolated from fibrotic organs maintain their ‘activated’ state even when removed from the stimulating environment [51–56] and in a similar way in our own cultured LC and TM cells from glaucomatous donors. Bechtel et al. hypothesised that epigenetic modifications may be a molecular cause for the activation of fibrotic fibroblasts and fibrosis[57]. They demonstrated that DNA methylation played a role in driving fibrosis in renal fibroblasts through TGFβ1 and Ras protein activator like 1 (RASAL1)[57]. RASAL1 is an inactivator of Ras, which drives cell proliferation when hyperactive, and this hyperactivity can be caused by loss of Ras-GTPase activating proteins (GAPs) such as RASAL1. RASAL1 was shown to have anti-fibrotic properties, and in the disease model, RASAL1 was downregulated directly by TGFβ1 and then indirectly by its promoter methylation through DNMT1 activity.

In glaucoma, there is evidence of a hypoxic environment[58–60]. A study by our laboratory demonstrated that LC cells subjected to hypoxia showed differential expression of genes involved in apoptosis, neurogenesis, ECM production, mitochondrion and angiogenesis[61]. A hypoxic environment has also been shown in studies that demonstrated the presence of hypoxia-inducible factor 1α (HIF1α) in the ONH; HIF1α is an indicator of hypoxia[58]. Hypoxia has been shown to induce an epigenetic response, which regulates the cellular response to the hypoxic insult in prostate cells[62]. DNA methylation has been shown to be increased by hypoxia in disease states[44, 62, 63]. Further, a recent study by Watson et al. showed that there was an association between increased collagen 1 and α-smooth muscle actin (αSMA) and hypoxia in human cardiac tissue. This was further associated with global DNA hypermethylation, and increased DNMT1 expression[63]. Therapeutically, there are DNMT inhibitors in current clinical use to treat myelodysplastic syndromes. 5-azacytidine (5-Aza) is one of these inhibitors and has been shown to ameliorate fibrosis in renal fibroblasts[57] and cardiac fibroblasts[64].

In our study, we wished to first examine the DNA methylation profile and the expression of TGFβ1, RASAL1 and HIF1α in primary GTM cells when compared to NTM cells. Subsequently, we wished to determine if hypoxia could induce a glaucomatous-like phenotype in NTM cells with regard to DNA methylation and TGFβ1 and RASAL1 expression. Further, we wished to establish the relationship between TGFβ1, RASAL1 and DNA methylation in TM cells in glaucoma. Our data show that GTM cells have different gene expression profiles compared to NTM cells with regards to TGFβ1 and RASAL1, and the enzymes that contribute to global DNA methylation. Exposure of NTM cells to hypoxia (1%O_2_) was shown to induce a similar phenotype to GTM cells with regard to DNA methylation as well as DNMT1, TGFβ1 and RASAL1 expression. We suggest that TGFβ1 and RASAL1 have an interacting role in glaucoma, which could perpetuate the associated fibrosis.

## Materials and Methods

### Cell Culture

Primary human donor trabecular meshwork cells were from normal (NTM) and glaucomatous (GTM) donors. Eyes were obtained from the Lion’s Eye Institute for Transplant and Research (LEITR), Tampa, Florida, United States of America (USA), donors or a first degree relative having given consent for their use for research purposes and TM cell explants from the same subsequently donated by Alcon Laboratories, Fort Worth, Texas, USA. The acquisition of these cells was carried out in accordance with the Declaration of Helsinki. The following TM cell strains were used: NTM160 (73yr old male), NTM210 (newborn, female), NTM444 (85 yr old male), NTM416 (78yr old male), GTM460 (77yr old male), GTM730 (88yr old male), GTM473 (86yr old male), GTM686 (71yr old female). The average age of the NTM donors (n = 4) was 52.67+/-52.67 yrs, and the GTM donors (n = 4) was 80.5+/-6.87 yrs (P = 0.36). These strains have been previously published[65, 66]. The cells were cultured at 37°C in 5% CO_2_ in Dulbecco's modified Eagle medium (DMEM) (D5546, Sigma, Ireland) supplemented with 10% (v/v) heat-inactivated foetal calf serum (FCS) (F9665, Sigma, Ireland), 10,000 units penicillin/mL and 10mg streptomycin/mL (P0781, Sigma, Ireland) and 4mM L-glutamine (G7513, Sigma, Ireland). Confluent TM cells were used between passages 3–8 for all experiments. Where indicated 3 normal and 3 glaucoma donor cell strains were used for each experiment or 3 independent experiments were performed using 3 NTM donor strains.

### Cell Hypoxia

To induce a hypoxic environment in the cells, NTM cells were cultured in full serum medium in a humidified hypoxia chamber (Coy Laboratories, USA) where the O_2_ was set to 1%, with 5%CO_2_ at 37°C for 6 and 24 hours as indicated. Induction of a hypoxic environment was confirmed by increased expression of HIF1α at both time points. Control cells were cultured in normoxic conditions (21%O_2_, 5%CO_2_, 37°C) for the same time points. The pH of medium from cells cultured under normoxic and hypoxic conditions was determined using a pH meter (Table 1). (n = 3)

**Table 1:** pH values of media from NTM cells subjected to hypoxia.

		pH Values	P-Value
6 hrs	Hypoxic TM Media	7.96+/-0.05	
6 hrs	Normoxic TM Media	7.99+/-0.49	P = 0.94
24 hrs	Hypoxic TM Media	7.97+/-0.21	
24 hrs	Normoxic TM Media	8.13+/-0.17	P = 0.44

### Cell Treatments

#### TGFβ1 Treatment

Cells were serum-starved for 24 hours before treatment. For TGFβ1 treatment, recombinant transforming growth factor β1 (T7039, Sigma, Ireland) was diluted to a concentration of 10ng/ml and added to NTM cells in culture for 24 hours. Following this, cells were processed for RNA or protein as described below. 3 independent experiments were performed.

#### 5-azacytidine Treatment

Cells were serum-starved for 24 hours before treatment. 5-Aza (A2385, Sigma, Ireland) was reconstituted in cell culture medium at a concentration of 0.5μM, this was then added to GTM cells in culture for 24 hours. 3 independent experiments were performed.

#### Hypoxia + 5-azacytidine Treatment

Cells were subjected to hypoxia for 6 and 24 hours, as described above, in the presence or absence of 5-azacytidine. 0.3μM 5-Aza was added to the cells for 24 hours under normoxic and hypoxic conditions as indicated. For the last 6 hours of the 5-aza treatment, cells were subjected to hypoxia (where 6 hours is indicated). Cells were co-treated with hypoxia and 5-aza for 24 hours (where 24 hours is indicated). 3 independent experiments were performed.

#### siRNA Treatment

Antibiotic free media with 10% FCS was used to treat NTM cells with small interfering RNA (siRNA) against TGFβ1. siRNA against TGFβ1 was added to NTM cells in culture at 10nM for 12, 24, 48 and 72 hours. 3 independent experiments were performed.

### Real-time quantitative PCR

Total RNA was isolated using TRIzol Reagent Solution (15596026, Life Technologies, Ireland) using the protocol suggested by the manufacturer. RNA was reverse transcribed to cDNA using AMV reverse transcriptase (A4464, Sigma, Ireland), oligo dT (O4387, Sigma, Ireland), and deoxynucleotides (dNTPs) (D7295, Sigma, Ireland). The value of the normal donors was set to an arbitrary value of one. 18S was used as a control to normalise Ct values, and the following genes were analysed using primers designed on qPrimerDepot and manufactured by Sigma: DNA methyltransferase (DNMT) 1 forward, 5'-AGCCCGTAGAGTGGGAATGGCA-3'; DNMT1 reverse, 5'-ACGCTTAGCCTCTCCATCGGACT-3'; Ras protein activator like 1 (RASAL1) forward, 5’-CGTGCTGGATGAGGACACTG-3’; RASAL1 reverse 5’-TCCCTGCTCAGCGAGATCTT-3’. qPCR primer-probes were also used for 18S (QF00530467, Qiagen, UK), DNMT3A (QF00427588, Qiagen, UK), Methyl-CpG binding protein 2 (MeCP2) (QF00138257, Qiagen, UK), TGFβ1 (QF00531146, Qiagen, UK), COL1A1 (QF00117607, Qiagen, UK) and αSMA (QF00531146, Qiagen, UK). A standard qPCR cycle was used consisting of: denaturation at 95°C for 10min, denaturation at 95°C for 10 sec, annealing at 55°C for 30 sec, and elongation at 72°C for 30 sec. This was repeated from the second denaturation step for 45 cycles followed by a final elongation step at 72°C for 5 min. The qPCR cycling protocol for DNMT3A was denaturation at 95°C for 10min, then denaturation at 95°C for 10 sec, annealing at 60°C for 30 sec, and elongation at 72°C for 20 sec. This was repeated from the second denaturation for 45 cycles followed by a final elongation step at 72°C for 5 min. Fold change in gene expression was assessed using the 2^Δ ΔCt^ equation in which the value of the normal donors is set to an arbitrary value of 1[67]. All qPCR results are shown as the mean fold change in gene expression of the experimental (treated/glaucoma) compared to control (untreated/normal) +/- standard deviation. (n = 3)

### Global Methylated DNA Quantification

Genomic DNA was isolated from confluent primary normal and glaucoma TM donor cells (n = 3) using a GenElute^™^ Mammalian Genomic DNA Miniprep Kit (G1N10, Sigma, Ireland) according to the manufacturer’s instructions. Briefly, the cells were lysed in a chaotropic salt-containing solution to ensure denaturation, and ethanol was used to precipitate DNA when the lysate was spun through a silica membrane. The DNA was then eluted in a Tris-EDTA solution (10mM Tris-HCl, 0.5mM EDTA, pH 9.0), and DNA concentration was determined by spectrometry (260nm). The equation (OD_260_ x 100(dilution factor) x 50μg/ml) was used to determine the DNA concentration. Gel electrophoresis was conducted on a 1% agarose gel to confirm the integrity of the eluted DNA.

Methylated DNA was quantified from the genomic DNA using the Imprint^®^ Methylated DNA Quantification Kit (MDQ1, Sigma, Ireland) using the protocol recommended by the manufacturer. Genomic DNA was bound to the wells and then an antibody for 5-methylcytosine was used to bind the methylated DNA, and a secondary detection antibody was used to create the colorimetric change that quantifies the level of methylated DNA. Each experiment was performed in triplicate. Results are presented as the mean value of optical density at absorbance 450nm +/-standard deviation.

### Western Blotting

Cells were subjected to hypoxia, or treated, as indicated and following treatment, the cells were scraped into ice-cold PBS followed by centrifugation to pellet the cells. The cells were resuspended in RIPA buffer (R0278, Sigma, Ireland) containing protease inhibitor cocktail (P8340, Sigma, Ireland), and then the cells were then incubated on ice for 5 mins and clarified by centrifugation as per the manufacturer’s instructions. Protein samples were added to sample buffer, boiled at 95°C for 5 mins, and stored at -80 ^o^C prior to electrophoresis. SDS-PAGE was then used to separate the proteins, and they were transferred to nitrocellulose membranes (N8267, Sigma, Ireland) and detected by immunoblotting according to standard protocols using ECL (10005943, Fisher Scientific, Ireland). β-actin was used as a loading control. Details of antibodies used are included in Table 2.

**Table 2:** Antibodies used for Western Blotting.

Target Protein	Host Species	Dilution	Product Code
TGFβ1	Rabbit	1:500	ab125287 (Abcam, UK)
HIF1α	Rabbit	1:500	3716 (Cell Signalling, USA)
DNMT1	Mouse	1:200	Sc271729 (Santa Cruz, Germany)
RASAL1	Mouse	1:400	ab168610 (Abcam, UK)
β-Actin	Rabbit	1:2000	4967 (Cell Signalling, USA)

### Cell Proliferation

Cells were seeded onto 96-well plates in triplicate. These were then treated as indicated. Following treatment, the CellTiter 96® AQueous One Solution Reagent (G3580, Promega, Ireland) was thawed and added to the wells as per the manufacturer’s protocol. The plate was incubated at 37°C in a humidified 5%CO_2_ environment for 1 hour. The absorbance was read at 490nm. Control readings were set to an arbitrary value of 100 and the experimental readings were set as a percentage of this.

### Statistical Analysis

Data are presented as the mean+/-standard deviation. The two-tailed unpaired Student’s t-test was used to analyse the statistical significance (*P<0.05, **P<0.01) of differences between mean values.

## Results

### Global DNA methylation was increased in GTM cells compared to NTM cells

**Fig 1:**
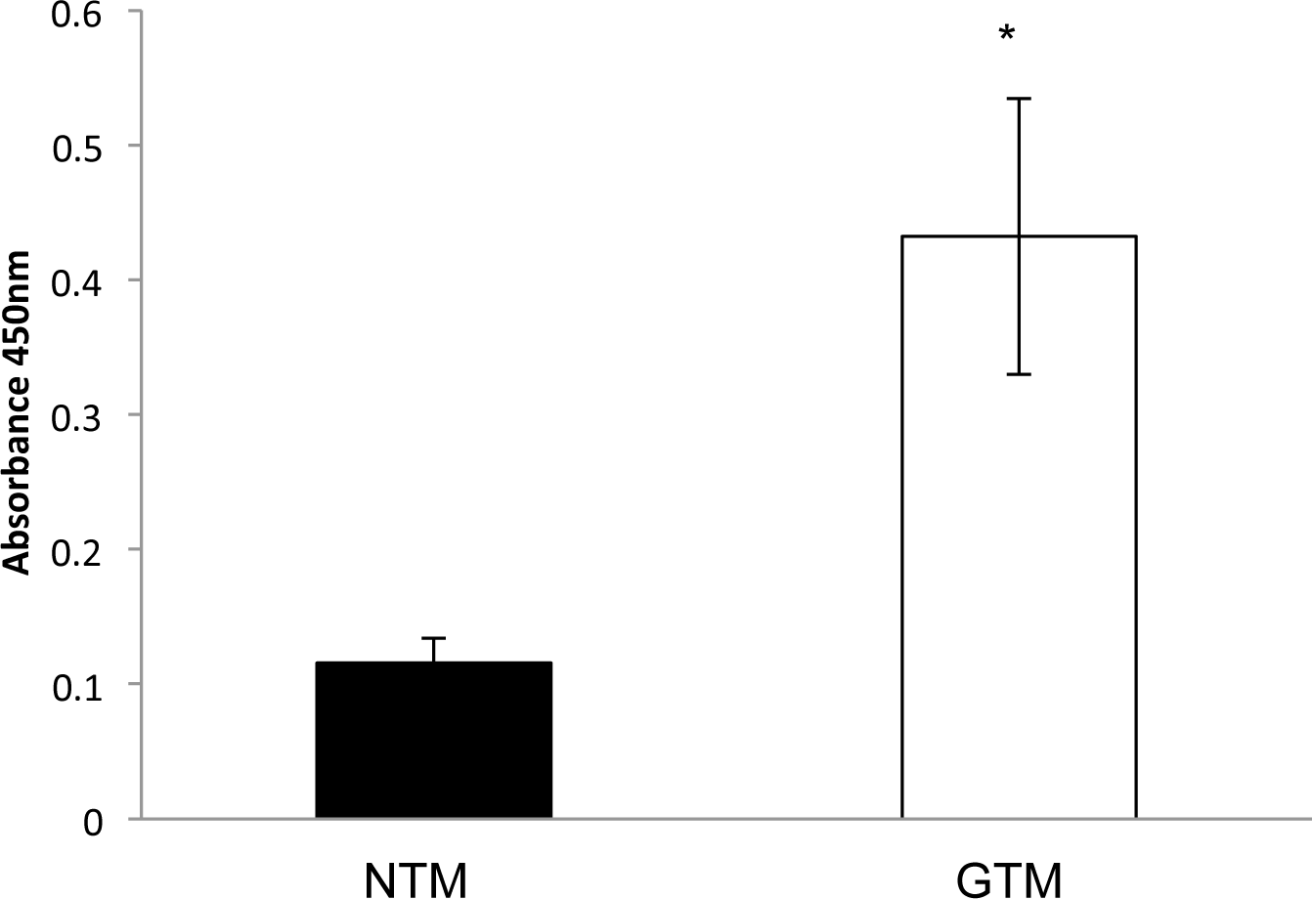
Global DNA methylation was increased in GTM cells compared to NTM cells. An ELISA assay was used to determine the level of DNA methylation in NTM and GTM cells. We found increased DNA methylation in the GTM cells compared to the NTM cells–NTM 0.12+/-0.02, GTM 0.43+/-0.1 (P<0.05). n = 3 *P<0.05. Results shown are from 3 normal and 3 glaucoma donors with each performed in triplicate.

We examined the methylation status of normal (NTM) and glaucomatous (GTM) trabecular meshwork cells. Global DNA methylation was examined by ELISA (Absorbance 450nm), and found to be signficantly (P<0.05) increased in GTM (Fig 1). Levels of DNA methylation in the cells were NTM: 0.12+/-0.02 and GTM: 0.43+/-0.1. (n = 3)

### Expression of TGFβ1 and RASAL1 by qPCR and Western blotting in GTM cells compared to NTM cells

**Fig 2:**
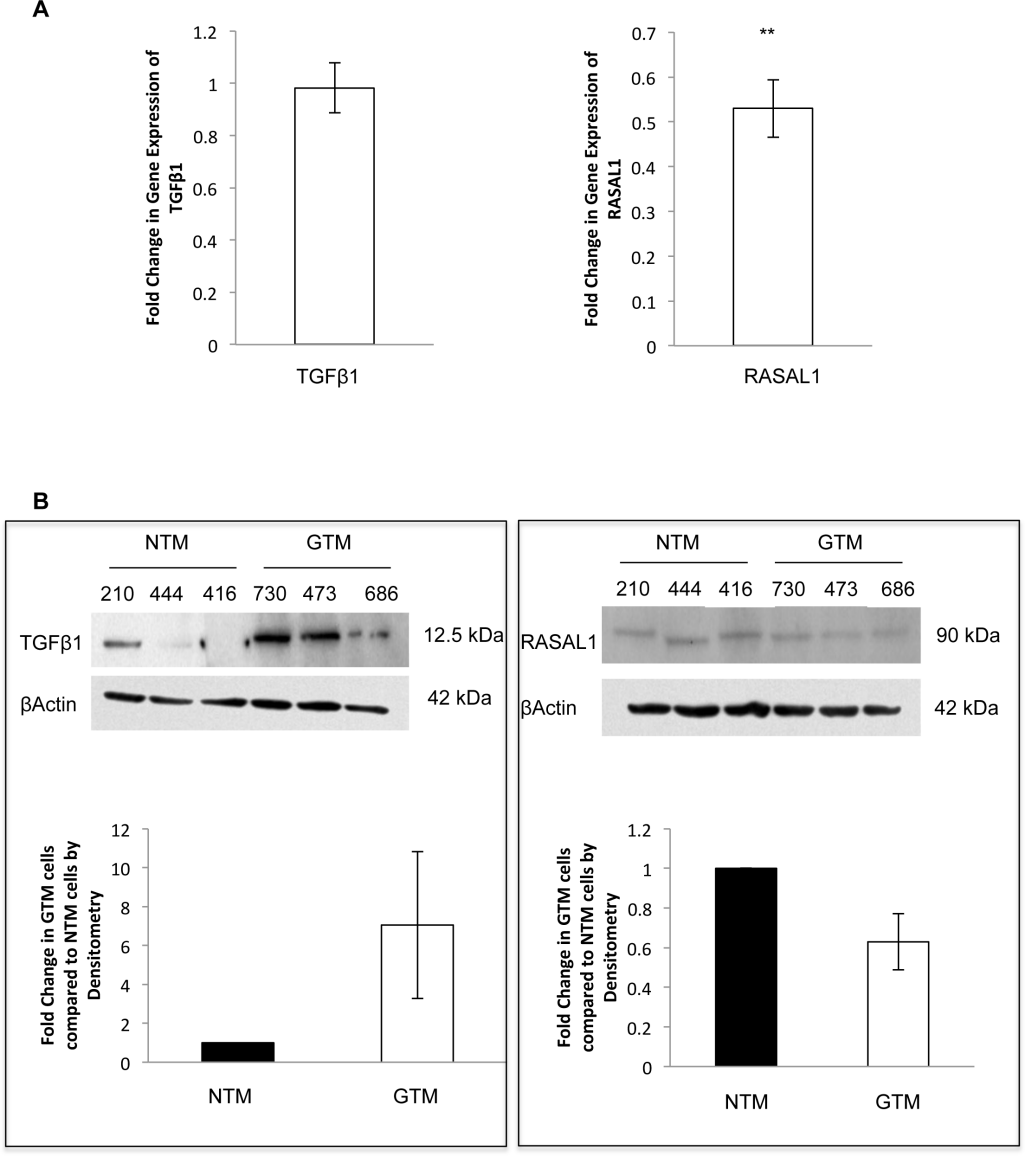
Expression of TGFβ1 and RASAL1 by qPCR and Western blotting in GTM cells compared to NTM cells. **A.** mRNA expression of TGFβ1 and RASAL1 by qPCR. There was no change in mRNA expression of TGFβ1 in GTM cells; 0.98+/-0.09 fold change. RASAL1 showed decreased mRNA expression in GTM cells; 0.53+/-0.06 (P<0.01). **B.** We examined the protein expression of TGFβ1 and RASAL1 by Western blotting and conducted densitometry on the blots. TGFβ1 protein expression was increased in GTM cells compared to NTM cells. RASAL1 showed decreased protein expression in GTM cells compared to NTM cells. n = 3 **P<0.01. Results shown are from 3 normal and 3 glaucoma donors with each performed in triplicate. (donor cell strain shown).

Upon examination of TGFβ1 and RASAL1 in TM cells by qPCR, we found that there was no change in TGFβ1 expression: fold change 0.98+/-0.09 (P<0.82) (Fig 2A). The expression of RASAL1 was significantly decreased in these cells: fold change 0.53+/-0.06 (P = 0.009) (Fig 2A). (n = 3)

We also examined TGFβ1 and RASAL1 protein expression by Western blotting (Fig 2B). We found that TGFβ1 protein appeared to be increased in the GTM cells compared to the NTM cells. Further, consistant with our mRNA data, RASAL1 protein expression also appeared to decrease in the GTM cells compared to the NTM cells. (n = 3)

### Expression of TGFβ1, COL1A1, DNMT1 and RASAL1 in GTM cells in response to the methylation inhibitor 5-azacytidine

**Fig 3:**
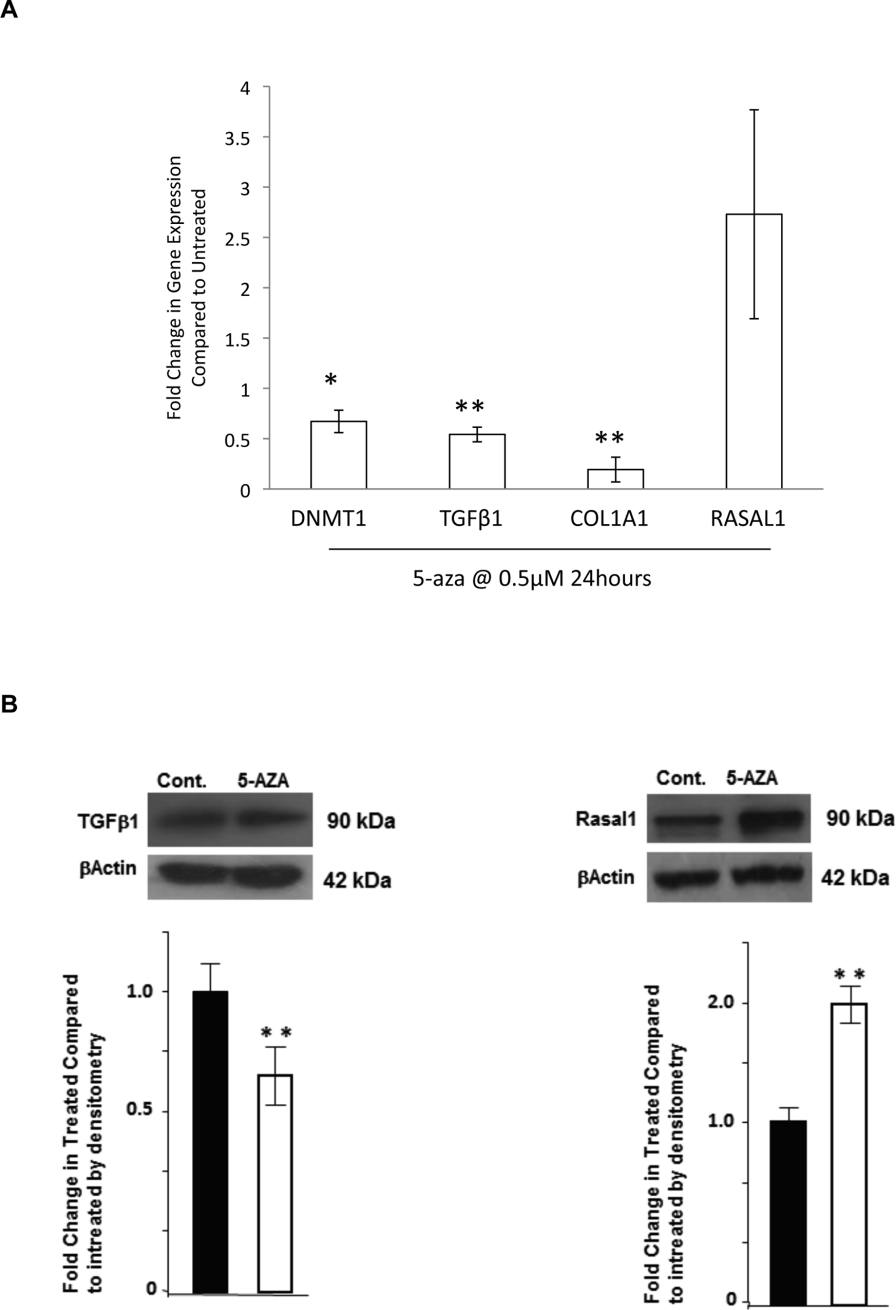
Expression of pro-fibrotic genes: TGFβ1, COL1A1, DNMT1 and RASAL1 in GTM cells in response to the methylation inhibitor 5-azacytidine. We examined the effect of the DNMT inhibitor 5-azacytidine on the mRNA expression of DNMT1, COL1A1, TGFβ1 and RASAL1 by qPCR (A). GTM cells were treated with 0.5μM 5-azacytidine for 24 hours. We found that DNMT1 was significantly (P<0.05) decreased by 5-azacytidine. Further, the pro-fibrotic genes COL1A1 and TGFβ1 were also significantly (P<0.01) decreased by 5-azacytidine. RASAL1 mRNA appeared to be increased in GTM cells treated with 5-azacytidine. n = 3 *P<0.05, **P<0.01 (B) Results for TGFβ1 and RASAL1 were confirmed at the protein level under identical experimental conditions. Control (Ctrl) GTM cells and GTM cells treated with 5-aza (0.5μM for 24 hours) were probed for TGFβ1 and RASAL1 by Western blot with βactin used as a loading control. Densitometry shows that there is a significant decrease in TGFβ1 (P<0.05) and increase in RASAL1 (P<0.05) expression levels following treatment. 3 independent experiments were performed. **P<0.05

To determine if altering DNA methylation by inhibiting DNMTs would ameliorate the increased expression of TGFβ1 and COL1A1 seen in GTM cells, we treated GTM cells with the DNMT inhibitor 5-aza (0.5μM) for 24 hours. This treatment was not cytotoxic to the cells (cell viability: ~97%). qPCR was used to examine gene expression of DNMT1, TGFβ1, COL1A1 and RASAL1 (Fig 3A). DNMT1 was decreased by 5-azacytidine (fold change 0.67+/-0.11 (P<0.05)). Further, TGFβ1 and COL1A1 were decreased in treated cells. Fold change in expression of TGFβ1; 0.54+/-0.07 (P<0.01) and COL1A1 0.19+/-0.12 (P<0.01). In contrast, RASAL1 expression appeared to increase in the 5-aza treated cells (fold change 2.73+/-1.04). (n = 3). Results for TGFβ1 (0.63+/-0.09) (P<0.05) and RASAL1 (1.98+/-0.13) (P<0.05) were confirmed at the protein level under identical experimental conditions (Fig 3B).

### HIF1α protein expression by Western blotting was increased in GTM cells and in NTM cells subjected to hypoxia

**Fig 4:**
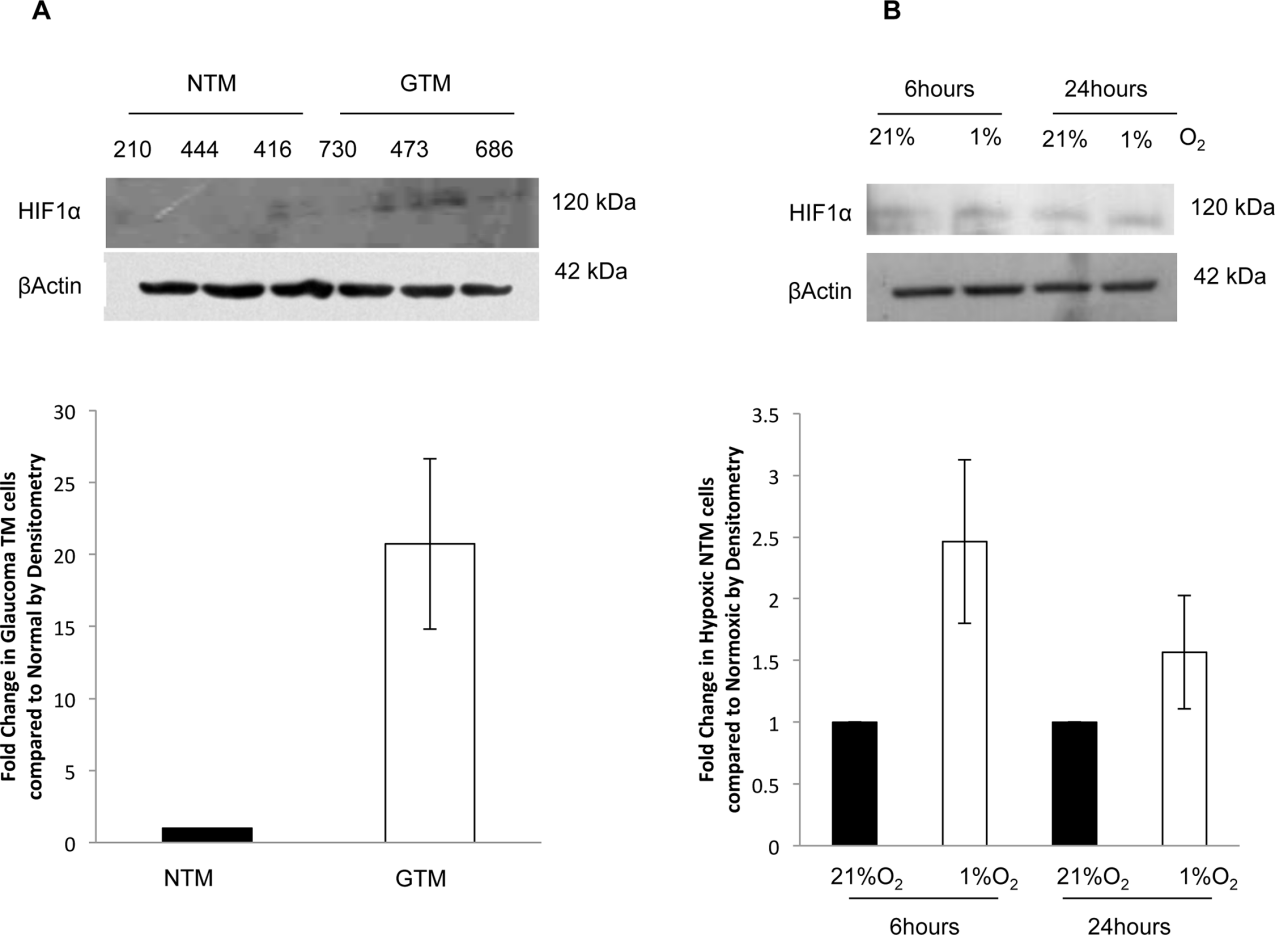
HIF1α protein expression by Western blotting was increased in GTM cells and in NTM cells subjected to hypoxia. Expression of HIF1α in glaucomatous TM cells compared to NTM cells, and in NTM cells subjected to 1%O_2_ by Western blotting. **A.** HIF1α expression was increased in GTM cells compared to NTM cells, this was reflected in densitometry conducted on the blots. 3 normal and 3 glaucoma donors used (donor cell strains indicated) **B.** A hypoxic environment (1%O_2_) was able to induce HIF1α expression in NTM cells at both 6 hours and 24 hours. This was also seen in the densitometry conducted on the blots. Results show data from 3 independent experiments.

To determine if there was an increase in HIF1α expression in GTM cells, we examined the expression of HIF1α by Western Blotting (Fig 4A). and found that there was increased expression of HIF1α in GTM cells compared to NTM cells as reflected in the densitometry conducted on the blots.

We wished to determine if a hypoxic (1%O_2_) environment could induce HIF1α expression in NTM cells after 6 and 24 hours (Fig 4B). We found that HIF1α was induced in NTM cells following hypoxia (1%O_2_) for 6 and 24 hours.

### Global DNA methylation and DNMT1 expression were increased in NTM cells subjected to hypoxia

**Fig 5:**
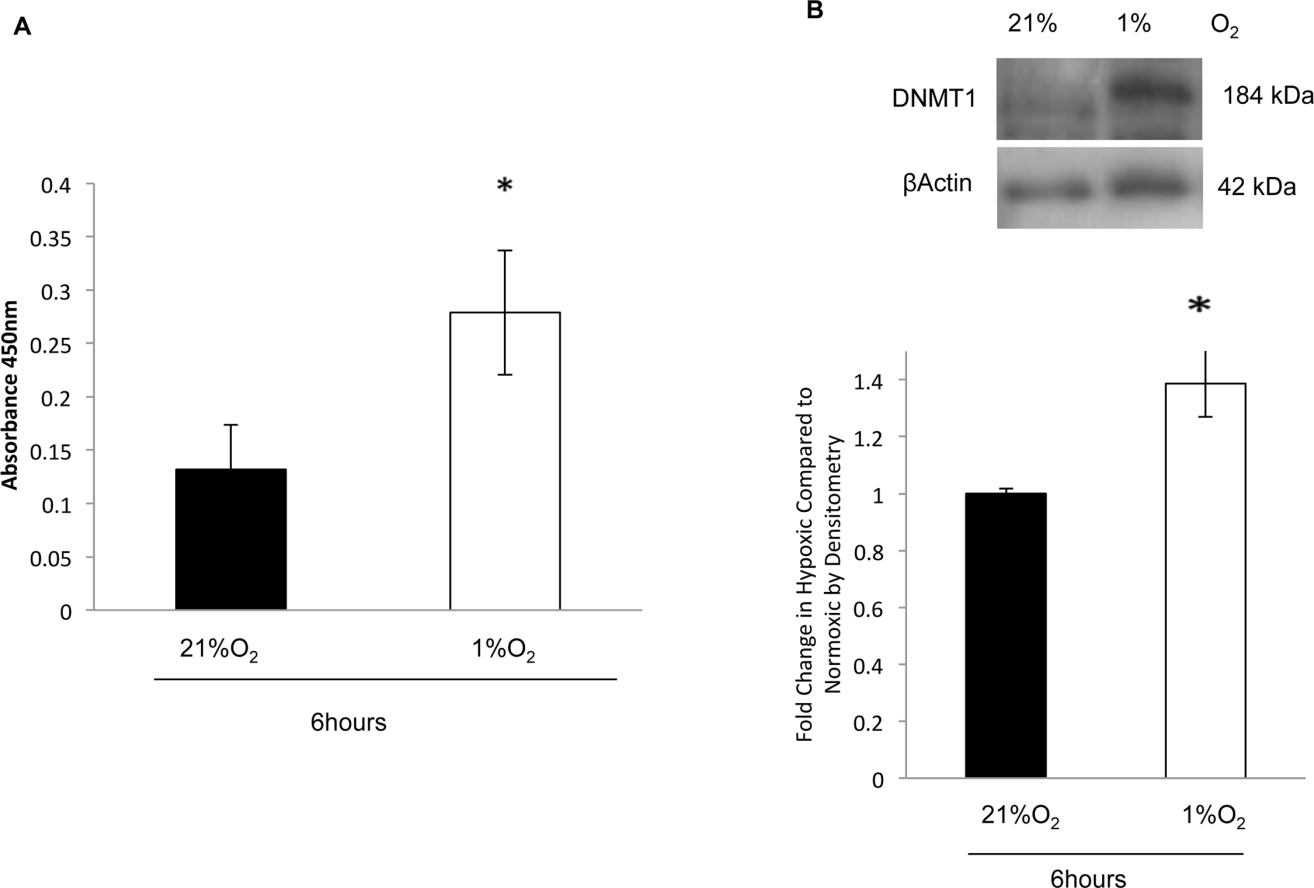
Global DNA methylation and DNMT1 expression were increased in NTM cells subjected to hypoxia. To determine if hypoxia was able to induce a glaucomatous-like phenotype in NTM cells. **A.** We examined global DNA methylation by ELISA. Global DNA methylation was increased in hypoxic TM cells (1% O_2_) and normoxic cells (21% O_2_) after 6 hours. Values were found to be 0.13+/-0.04 (21%O_2_), 0.28+/-0.06 (1%O_2_) (p<0.05). **B.** We examined protein expression of DNMT1 by Western Blotting in NTM cells subjected to hypoxia, and conducted densitometry on the blots. We found a significant increase in DNMT1 expression after 6 hours hypoxia. n = 3 *P<0.05

We subjected NTM cells to hypoxia (1%O_2_) to determine if this could induce a glaucomatous-like methylation status phenotype. As before, we examined global DNA methylation by ELISA, mRNA expression by qPCR and protein expression of DNMT1 by Western blotting. Global DNA methylation was increased in cells subjected to hypoxia after 6 hours. The levels of DNA methylation were; NTM: (21%O_2_) 0.13+/-0.04 and NTM (1%O_2_): 0.28+/-0.06 (P<0.05) (Fig 5A). (n = 3). DNMT1 expression was significantly increased following 6 hours under hypoxic conditions (Fig 5B). This was reflected in the densitometry analysis of three independent blots (P<0.05) (Fig 5B). (n = 3)

### TGFβ1 expression was increased, and RASAL1 expression was decreased in NTM cells in response to a hypoxic environment

**Fig 6:**
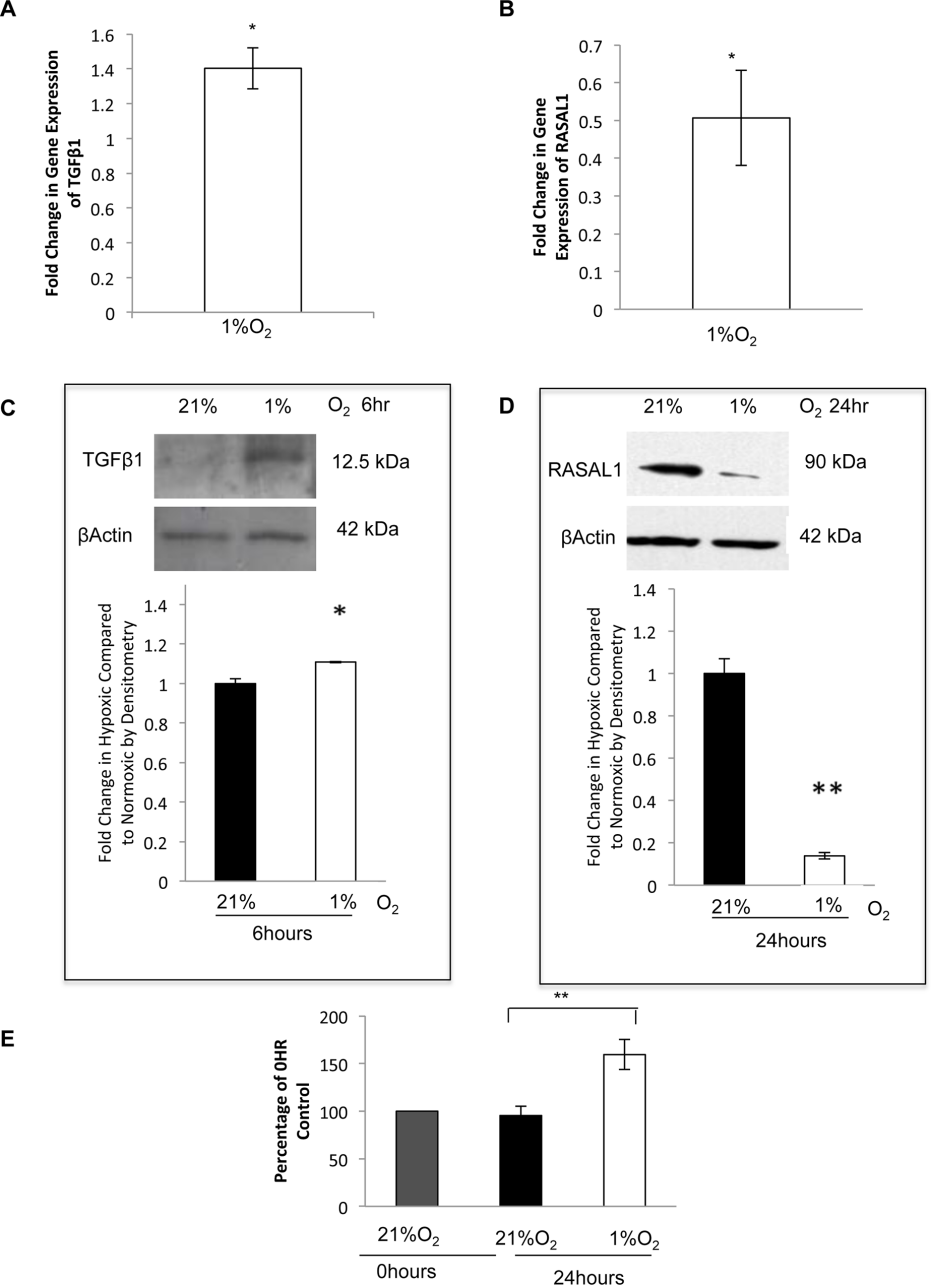
TGFβ1 expression was increased, and RASAL1 expression was decreased in NTM cells in response to a hypoxic environment. We examined mRNA expression of TGFβ1 and RASAL1 by qPCR and protein expression by Western blotting under normoxic (21%O_2_) and hypoxic conditions (1%O_2_). **A.** qPCR showed an apparent increase in TGFβ1 mRNA expression in cells subjected to hypoxia after 6 hours; fold change in gene expression was 1.37+/-0.16. (P<0.05) **B.** After 24 hours, RASAL1 was decreased; fold change 0.51+/-0.13 (P<0.05). **C.** Western blotting shows an apparent increase in TGFβ1 after 6 hours. **D.** After 24 hours, RASAL1 protein was decreased (P<0.06). **E.** An MTS assay was conducted to determine cell proliferation in NTM cells subjected to 1%O_2_ for 24 hours. A 0hr Control was set to 100%, and the normoxic and hypoxic results were presented as a percentage of this; Normoxic = 95.5%, Hypoxic = 159.4% (P<0.01). n = 3 *P<0.05, **P<0.01. Results are from 3 independent experiments.

To determine if hypoxia (1%O_2_) could increase TGFβ1 and decrease RASAL1 in NTM cells, similar to that observed in GTM cells we examined the mRNA and protein expression of both TGFβ1 and RASAL1 in NTM cells subjected to hypoxia. After 6 hours hypoxia, TGFβ1 mRNA expression appeared to increase (fold change: 1.37+/-0.16) (P<0.05) in hypoxic cells (Fig 6A), this was reflected in TGFβ1 protein expression (Fig 6C).

RASAL1 mRNA expression showed a significant decrease after 24 hours (fold change: 0.51+/-0.13 (P<0.05)) (Fig 6B). This was also apparent at the protein level, RASAL1 showed an initial increase in expression after 6 hours which was then decreased after 24 hours (Fig 6D).

We examined cell proliferation as Ras is a regulator of this activity, and when RASAL1 is downregulated, Ras becomes more active, possibly increasing cell proliferation. Cell proliferation in NTM cells was found to be increased following 24 hours of hypoxia (Fig 6E). A 0hr control was used to determine baseline proliferation, cells were then left at normoxic cell culture conditions as above, or subjected to hypoxia, as above. 0hr control was set to 100%, NTM cells under normoxic conditions were 95.5% compared to the 0hr control, and the NTM cells subjected to hypoxia showed increased proliferation of 159.4% compared to the 0hr control. Statistical analysis for the normoxic and hypoxic results and showed significance of P<0.01.

### TGFβ1 and COL1A1 expression was decreased and RASAL1 expression was increased by Western blotting in NTM cells under both normoxic and hypoxic conditions

**Fig 7:**
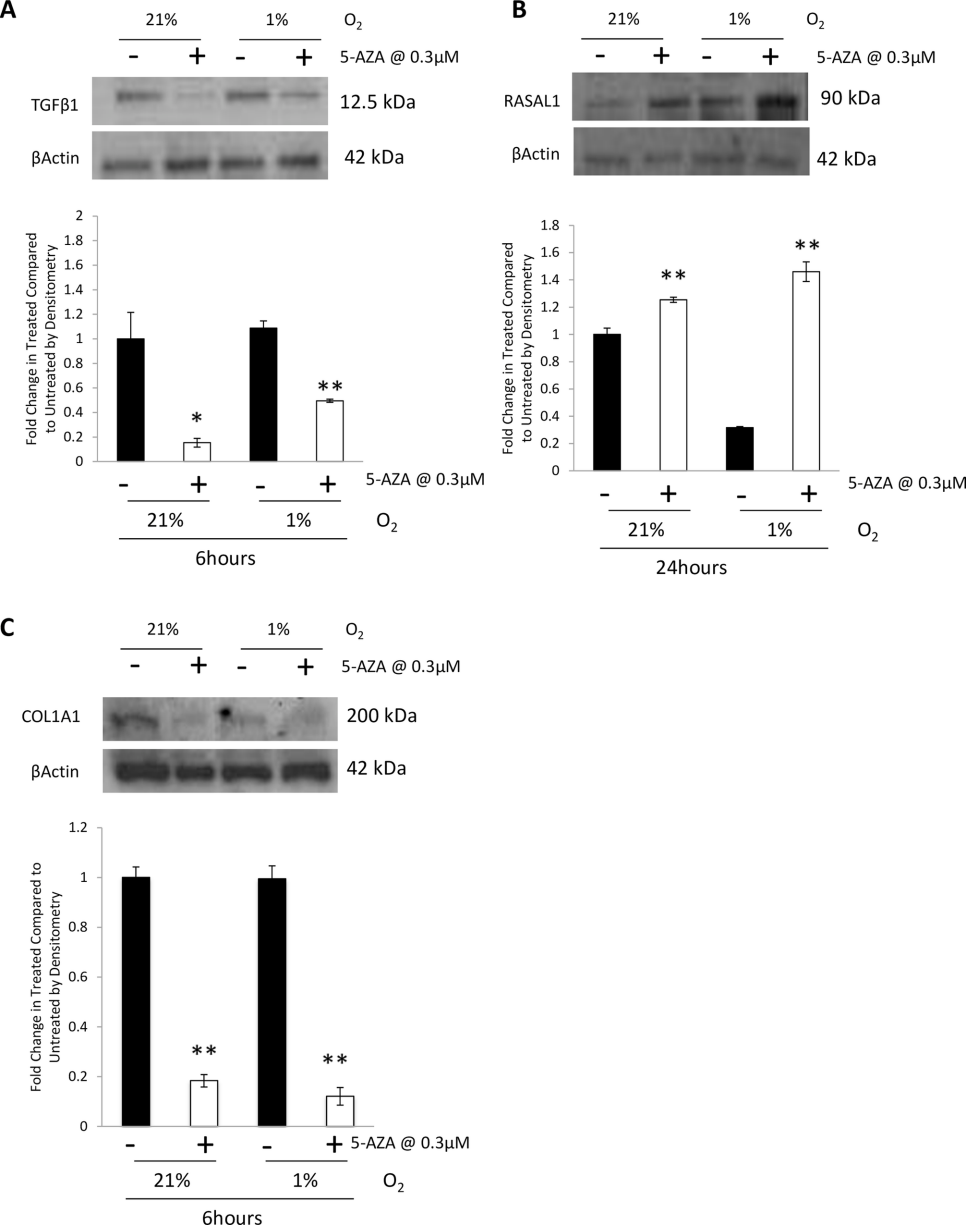
TGFβ1 and COL1A1 expression was decreased and RASAL1 expression was increased by Western blotting in NTM cells under both normoxic and hypoxic conditions. We examined the effect of the DNMT inhibitor 5-azacytidine on TGFβ1 and RASAL1 expression in NTM cells under hypoxic conditions. A. NTM cells were treated with 0.3μM 5-azacytidine for 24 hours and then subjected to 1%O_2_ for the last 6 hours of treatment. We found that TGFβ1 protein was decreased in the presence of 5-aza.B. NTM cells were treated with 0.3μM 5-aza for 24 hours and subjected to 1%O_2_. We found that RASAL1 was increased in the presence of 5-aza. C. NTM cells were treated with 0.3μM 5-aza for 24 hours and then subjected to 1%O_2_ for the last 6 hours of treatment. We found that COL1A1 protein was decreased in the presence of 5-aza. One representative blot is presented. We also show densitometry analysis of the blots. *P<0.05, **P<0.01 Results are from 3 independent experiments.

We examined the effect of 5-azacytidine (0.3μM) on the expression of TGFβ1, RASAL1 and COL1A1 in NTM cells subjected to hypoxia by Western blotting. After 24 hours 5-aza treatment and 6 hours hypoxia, TGFβ1 was decreased in NTM cells treated with 5-aza. Analysis of the blots by densitometry showed a significant decrease in TGFβ1 expression (P<0.05, P<0.01) (Fig 7A).

After 24 hours 5-aza and 24 hours hypoxia, RASAL1 expression showed an increase in protein expression in the presence of 5-aza, and this was also seen in the analysis of the blots by densitometry (Fig 7B).

After 24 hours 5-aza treatment and 6 hours hypoxia, COL1A1 was decreased in NTM cells treated with 5-aza. Densitometry analysis of the blots showed a significant decrease in the expression of COL1A1 with 5-aza treatment (P<0.01) (Fig 7C).

### NTM cells treated with TGFβ1 show increased expression of COL1A1 and DNMT1 and decreased expression of RASAL1

**Fig 8:**
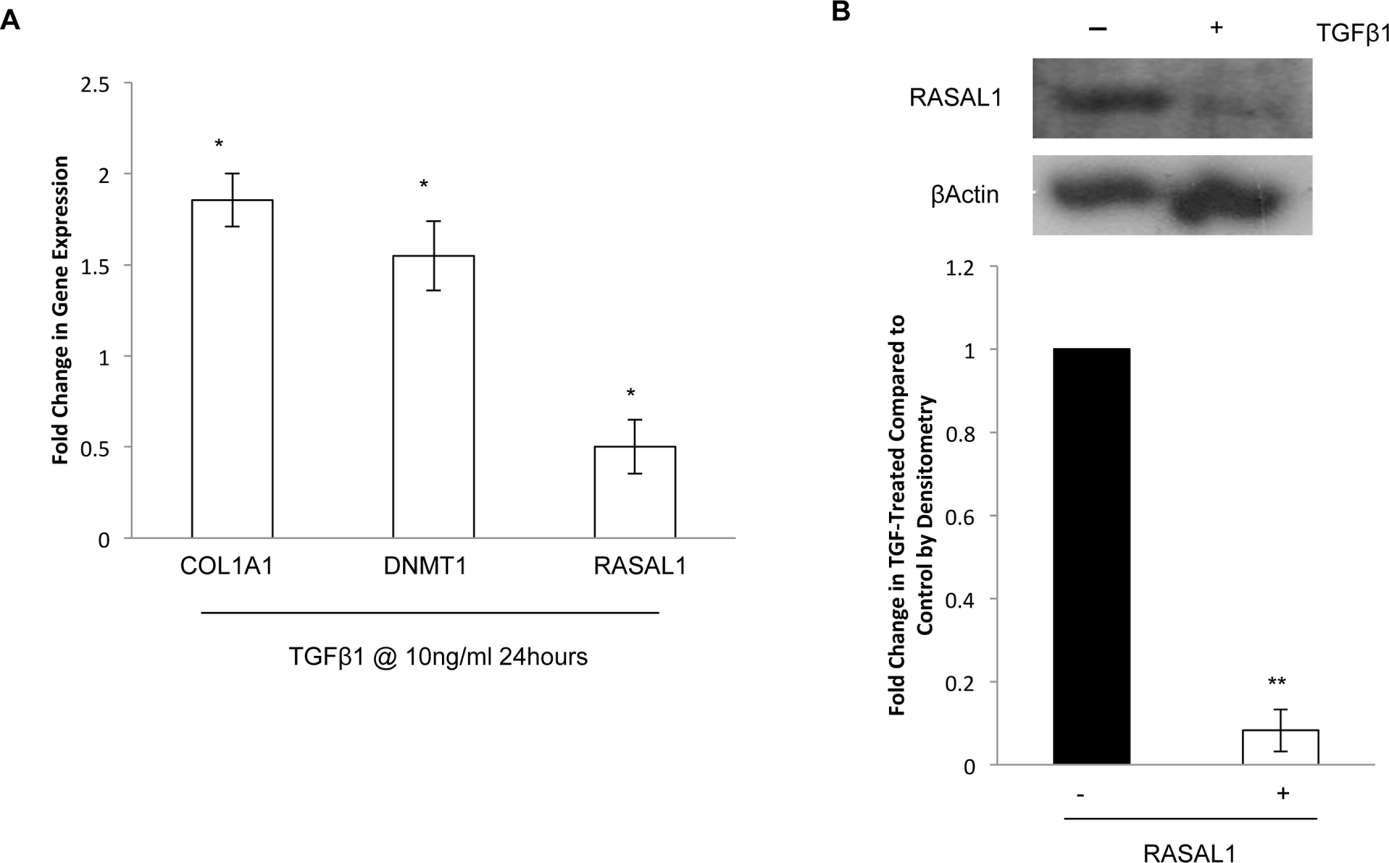
NTM cells treated with TGFβ1 show increased expression of COL1A1 and DNMT1 and decreased expression of RASAL1. We examined the effect of 10ng/ml TGFβ1 on NTM cells after 24 hour treatment. **A.** There was no significant change in cell viability of cells treated with 10ng/ml TGFβ1. **B.** Upon investigation of mRNA expression of COL1A1, DNMT1 and RASAL1 by qPCR, we found that COL1A1 (P<0.05) and DNMT1 were increased and RASAL1 (P<0.05) was decreased in the cells treated with TGFβ1. **C.** We examined RASAL1 protein by Western blotting and conducted densitometry on the blots. We found that, like the mRNA expression, TGFβ1 decreased RASAL1 expression in NTM cells. *P<0.05, **P<0.01 Results are from 3 independent experiments.

To determine the effect of TGFβ1 on DNMT1 and RASAL1, NTM cells were treated with TGFβ1(10ng/ml) for 24 hours, we conducted qPCR for the expression of collagen 1a1 (COL1A1), DNMT1 and RASAL1 expression (Fig 8A). We found that TGFβ1 increased COL1A1 (1.85+/-0.14) and DNMT1 (1.55+/-0.19) significantly (P<0.05). Treatment of NTM cells with TGFβ1 significantly decreased RASAL1 expression (P<0.05) (fold change 0.5+/-0.15). We also examined RASAL1 protein by Western blotting to determine if the decrease in RASAL1 occurred at the protein level, and found that RASAL1 protein was also decreased by TGFβ1 treatment (Fig 8B) (P<0.01).

### siRNA knockdown of TGFβ1 in NTM cells led to an increase in RASAL1 expression and a decrease in COL1A1 and DNMTI expression.

We had established that treatment of cells with TGFβ1 could decrease the expression of RASAL1, and that a methylation inhibitor could increase RASAL1 expression, we next wanted to determine if knocking down TGFβ1 could also increase RASAL1 expression.

We used siRNA for TGFβ1 to knockdown its expression in NTM cells. Previous siRNA treatments in NTM cells demonstrated that 10nM siRNA was an effective dose[68], we therefore used this dose in our experiments. The cells were treated with siRNA for 12, 24, 48 and 72 hours and Western blotting was conducted to determine the expression of TGFβ1, phosphorylated Smad 3 (p-Smad3), total Smad 3 and RASAL1.

**Fig 9:**
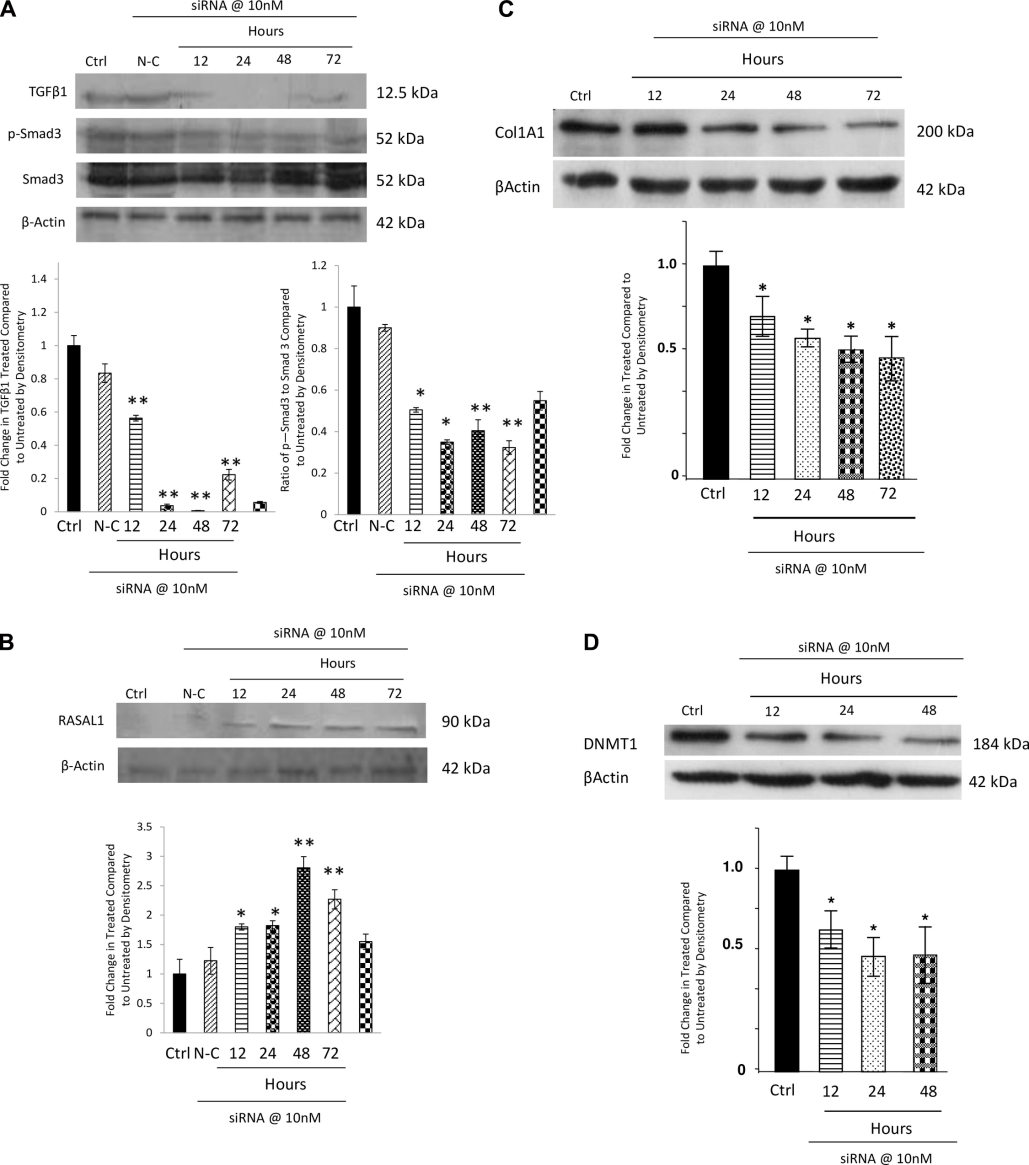
siRNA knockdown of TGFβ1 in NTM cells led to an increase in RASAL1 expression by Western blotting. We examined the effect of knocking down TGFβ1 expression in NTM cells using 10nm siRNA for TGFβ1. A. siRNA for TGFβ1 decreased the protein expression of TGFβ1, p-Smad3 and Smad3. This indicates that when TGFβ1 is decreased, its pathway is also affected. Further, a decrease in TGFβ1 expression by siRNA treatment also led to an increase in RASAL1 protein expression B, a decrease in COL1A1 expression C and a decrease in DNMT1 expression D. Ctrl–Untreated Cells, N-C–Non-coding siRNA (negative control). We show one representative blot. We also show densitometry analysis of the blots. n = 3 *P<0.05, **P<0.01

We found that siRNA for TGFβ1 was able to decrease TGFβ1 protein expression and decrease the expression of p-Smad3, while total Smad3 remained unchanged, indicating that the signalling pathway was also affected by the siRNA treatment (Fig 9A). Protein expression of RASAL1 was increased when TGFβ1 expression was decreased by the siRNA (Fig 9B).

Furthermore, we also examined the effect of knockdown of TGFβ1 on expression of COL1A1 and DNMT1 by Western Blotting (Fig 9C&9D). Here, we found that expression of COL1A1 is also reduced as TGFβ1 expression decreases (P<0.05) as is DNMT1 (P<0.05).

## Discussion

**Fig 10:**
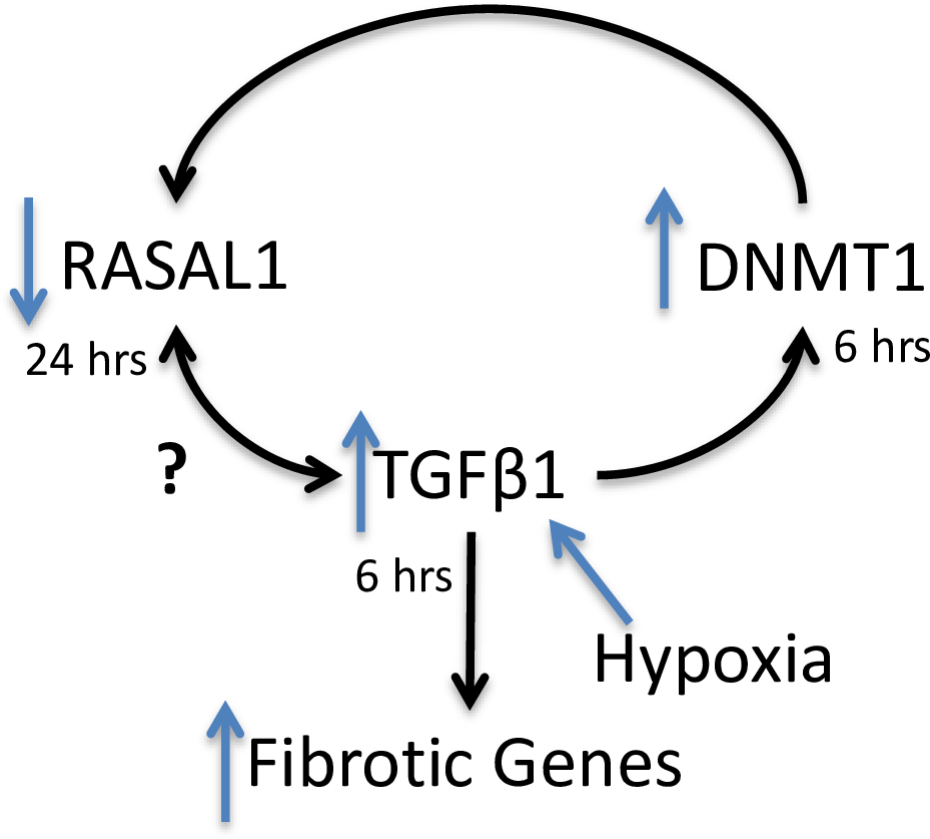
Hypoxia induces altered DNA methylation which alters the regulation of TGFβ1 and RASAL1. There is a previously established hypoxic environment in glaucoma[58]. Our hypothesis is that hypoxia increases TGFβ1 expression [70] and alters the DNA methylation profile of cells[44, 71]. TGFβ1 has a HRE which allows it to be regulated by HIF1α, and hypoxia[72]. TGFβ1 has been shown to upregulate DNMT1 expression in fibroblasts[57]. Furthermore, TGFβ1 has been shown to downregulate RASAL1 directly, and has been shown to induce promoter hypermethylation of RASAL1 thorough DNMT1 in mouse renal fibroblasts[57]. However it has yet to be established if RASAL1 can also regulate TGFβ1.

Increased IOP is the only current treatment for glaucoma[17]; however, this is only one of the risk factors for glaucoma and there are no current treatments in clinical use to target other risk factors or mechanisms driving the disease. One such mechanism is the fibrosis associated with the trabecular meshwork and lamina cribrosa regions. We hypothesise that the hypoxic environment seen in glaucoma[69] may contribute to the disease fibrosis by changing the global methylation profile of the cells, which subsequently alters the expression of TGFβ1 and RASAL1 (Fig 10). We show that there is an altered DNA methylation status, increased TGFβ1 and decreased RASAL1 in GTM cells compared to NTM cells. Further, we used hypoxia as a stimulus to determine if it could induce a glaucomatous-like DNA methylation status and similar expression profiles of TGFβ1 and RASAL1 in NTM cells. We found that hypoxia was sufficient to alter the DNA methylation status of the cells, and that it also increased TGFβ1 and decreased RASAL1 in the NTM cells.

We examined global DNA methylation and found GTM cells to possess significantly higher levels of methylation compared with NTM cells. Changes in DNA methylation have been shown to play a role in driving fibrosis in other diseases and disease models[44, 57, 73]. This may be occurring in glaucoma to drive the fibrotic processes that contributes to disease progression. There is wide variation in the level of DNA methylation between different donor cells, consistent with evidence for each person having their own epigenetic profile[74, 75]. Examination of TGFβ1 and RASAL 1 expression in GTM cells by qPCR showed no change in expression of TGFβ1 in GTM cells compared to NTM cells and that RASAL1 was significantly decreased in the GTM cells compared to the NTM cells. TGFβ1 protein was increased in GTM cells and RASAL1 protein was decreased in GTM cells. This may suggest that there is an inverse relationship between the expression of TGFβ1 and RASAL1 in the GTM cells. Although the mRNA expression of TGFβ1 showed no change, we found an increase in the protein expression of this cytokine. This may be because mRNA expression is not always indicative of protein expression, due to post-translational modifications, feedback loops and the half-life of the protein, the expression of mRNA and protein can differ[76, 77]. We also found some variation in the expression of TGFβ1 in NTM donors; this could be due to other undocumented underlying diseases affecting these patients

Results showed increased HIF1α expression in GTM cells compared to NTM cells. To determine if hypoxia is one of the drivers of glaucoma *in vivo*, we subjected NTM cells to a 1%O_2_ environment to induce hypoxia in the cells for 6 hours and for 24 hours. 1%O_2_ has previously been shown to induce hypoxia in LC and RGC cells[61, 78], and this level of oxygen has been used as a stimulus to induce hypoxia in other systems including endothelial cells[79] and HeLa cells[80]. We then determined that this level of oxygen increased HIF1α expression in NTM cells.

Examination of the DNA methylation status of NTM cells under hypoxic conditions was performed to determine if it was similar to that observed in GTM cells. We found that hypoxia changed the global methylation and the expression of DNMT 1. These changes were similar to those we obtained in the GTM cells where we observed an increase in global DNA methylation.

Hypoxia appeared to increase expression of TGFβ1 and significantly decreased RASAL1 expression in NTM cells. The TGFβ1 promoter contains a hypoxia response element (HRE) that allows it to be regulated by hypoxia[72]. Upon examination of the mRNA and protein expression of TGFβ1 and RASAL1. We found that TGFβ1 protein was increased after 6 hours hypoxia, while RASAL1 protein expression was decreased after 24 hours. We believe this is a similar mechanism to that seen in mouse kidney fibroblasts by Bechtel et al[57]. They reported that TGFβ1 was able to directly decrease RASAL1 expression after 24 hours, and that decreasing RASAL1 expression led to activated, fibrotic fibroblasts. Further, there was significantly increased cell proliferation in NTM cells subjected to hypoxia for 24 hours, which correlates with the decrease in RASAL1 expression. RASAL1 is a regulator of Ras, which is associated with cell proliferation[81]. This demonstrates that hypoxia does induce a glaucomatous-like phenotype in NTM cells.

As mentioned, RASAL1 is an inactivator of Ras, which drives cell proliferation when hyperactive. Ras hyperactivity can be caused by loss of Ras-GTPase activating proteins (GAPs) such as RASAL1. It has been previously established that knockdown of RASAL1 expression by methylation in nonfibrotic mouse renal fibroblasts induced the same cell phenotype as activated, fibrotic mouse renal fibroblasts[57]. RASAL1 has previously been demonstrated to be decreased in fibrosis[57, 82]. Furthermore, TGFβ1 was capable of decreasing RASAL1 expression, both directly and indirectly through inducing DNMT1, which causes promoter hypermethylation[57]. We investigated the relationship between DNA methylation, TGFβ1 and RASAL1 by treating NTM cells with TGFβ1, and by treating GTM cells with the DNMT inhibitor 5-azacytidine. The mRNA expression of COL1A1 and DNMT1 were significantly increased by TGFβ1, while RASAL1 was decreased. RASAL1 protein was also decreased by TGFβ1 treatment. This demonstrates that TGFβ1 drives a pro-fibrotic phenotype in NTM cells, and that this was done, in part, through down regulation of RASAL1 possibly by promoter methylation. Treatment of GTM cells with 5-aza showed that DNMT1, TGFβ1 and COL1A1 expression were decreased. There was also a significant increase in RASAL1 indicating that decreasing DNMT activity may ameliorate fibrosis in glaucoma, and that this may be through the RASAL1 pathway. We also demonstrated that siRNA knockdown of TGFβ1 and phosphorylated Smad 3 in normal TM cells, also correlated with an increase in RASAL1 expression and a resultant decrease in expression of COL1A1 and DNMT1 This further demonstrates that there is an inverse correlation between TGFβ1 and RASAL1 expression in trabecular meshwork cells, and that TGFβ1 may play a role in regulating RASAL1 expression as seen in renal fibrosis[57] perhaps in a similar methylation-dependent manner.

DNA methylation was increased in both glaucoma TM cells, and in normal TM cells subjected to hypoxia, and 5-aza was able to ameliorate the increased TGFβ1 and decreased RASAL1 observed in glaucoma TM cells. 5-aza also decreased the expression of the pro-fibrotic TGFβ1 and increased RASAL1 (which has anti-fibrotic properties) in normal TM cells under both normoxic and hypoxic conditions. As this inhibitor is clinically used to treat myelodysplastic syndromes[83] it may be a potential treatment for reducing the fibrosis seen in glaucoma. 5-aza has previously been shown to decrease fibrosis in kidney fibroblasts and renal fibrosis[57] [82]. Bechtel et al showed decreased COL1 and αSMA in fibrotic kidney fibroblasts treated with 5-aza [57]. The accumulation of αSMA and fibroblast-specific protein 1 (FSP1) was decreased in kidney sections of mice treated with 5-AZA compared to untreated mice[82]. 5-aza has also been shown to inhibit fibrosis in a model of myocardial infarction (MI)[64]. Over all, these studies indicate that 5-aza can reduce fibrosis *in vitro* and *in vivo* in disease models.

Interestingly, we found that 5-aza also decreased TGFβ1 expression and increased RASAL1 expression in normal TM cells under normoxic conditions. DNA is normally methylated to a certain extent in healthy subjects. Therefore, it is likely that 5-aza would affect the global methylation levels in these cells. Further, the expression of RASAL1 has been shown to be regulated by promoter methylation[57, 82, 84]. We believe that TGFβ1 and RASAL1 may have a reciprocal relationship in which they play a role in regulating each other. As 5-aza restores the expression of RASAL1, it may also result in the decreased expression of TGFβ1 under both normoxic and hypoxic conditions.

In conclusion, we have shown increased global DNA methylation in both GTM cells, and NTM cells subjected to hypoxia, accompanied by altered gene expression of DNMT1. We have also shown an increase in the pro-fibrotic cytokine TGFβ1 and a decrease in RASAL1, which has have anti-fibrotic properties, in GTM cells and NTM cells subjected to hypoxia. We also investigated the association of TGFβ1, RASAL1 and DNMT1, which may drive the increase in expression of profibrotic genes seen in the glaucomatous TM.
